# Validation and psychometric analysis of the Internet Addiction Test in Spanish among college students

**DOI:** 10.1186/s12889-015-2281-5

**Published:** 2015-09-24

**Authors:** Tania Fernández-Villa, Antonio J. Molina, Miguel García-Martín, Javier Llorca, Miguel Delgado-Rodríguez, Vicente Martín

**Affiliations:** The Research Group in Gene - Environment and Health Interactions, University of León, León, Spain; Faculty of Health Sciences, Department of Biomedical Sciences, Area of Preventive Medicine and Public Health, University of León, Vegazana Campus s/n, 24071 León, Spain; Department of Preventive Medicine and Public Health, University of Granada, Granada, Spain; The Biomedical Research Centre Network for Epidemiology and Public Health (CIBERESP), Madrid, Spain; Area of Preventive Medicine and Public Health, University of Cantabria, Cantabria, Spain; Area of Preventive Medicine and Public Health, University of Jaén, Jaén, Spain

**Keywords:** Internet addiction, Internet addiction test, Factorial validity, College students, Spain

## Abstract

**Background:**

The wide use of the Internet in the workplace, academic or social field, can have an impact on daily life. One of the most used questionnaires worldwide to analyse these problems is the Internet Addiction Test (IAT). Our aim was to validate a Spanish version of the IAT and analyse its psychometric properties.

**Methods:**

Population of study were college students participating in the uniHcos project (Universities of Granada, Huelva, Jaén, León, Salamanca, and Vigo). The questionnaire was translated and back-translated by two native English speakers. Reliability of scores was analysed using Pearson’s correlation coefficient and agreement was analysed using the Bland-Altman and Kappa techniques. Test dimensions were analysed by exploratory and confirmatory factor analysis.

**Results:**

The reliability of scores was good (*r* = 0.899, Kappa = 0.650 and mean difference using Bland-Altman = −3.5). The psychometric assessment identified two factors (Emotional Investment; Performance and Time Management) which explained 55 % of the variance (total internal consistency of 0.91) and only 19 items. The confirmatory analysis showed an acceptable goodness of fit, especially when items 6 and 8 were related (RMSEA = 0.07 90%IC = 0.06 - 0.08; WRMR = 1.01, CFI = 0.96; TLI = 0.95). The two dimensions were negatively correlated with age and positively correlated with time spent online, especially for the purposes of leisure and entertainment.

**Discussion:**

The results show good reliability and psychometric properties of the Spanish version of IAT with a two-dimensional solution. This result is partially in concordance with previous validations of the IAT in other languages that have found uni- and multi-dimensional solutions using different methodologies. Moreover, we want to highlight the possibility that some item of this questionnaire is outdated due to the technological and lifestyles changes and should be not taken into account.

**Conclusion:**

The reliability and psychometric properties obtained in this study support the conclusion that this Spanish short version of the IAT represents a useful tool for the analysis of problems arising from misuse of the Internet.

**Electronic supplementary material:**

The online version of this article (doi:10.1186/s12889-015-2281-5) contains supplementary material, which is available to authorized users.

## Background

The Internet has become an everyday tool for finding information and communicating in both academic and professional contexts, as well as being a medium for socialising and entertainment, especially among young people [1, 2].

According to the latest Survey on Equipment and Use of Information and Communication Technologies in Households in Spain, 74 % of households have Internet access, representing 22 % more than five years ago. The 71 % of the population aged between 16 and 74 uses the Internet frequently (at least once a week in the last three months) and 60 % has heavy use (every day) of this medium [2].

However, despite being a useful tool in our daily lives, it can also have negative consequences, especially when users exhibit a lack of self-control regarding the length of time they spend online, which can be related to states of Internet use, abuse or dependence, being the most vulnerable groups the adolescents and young adult [3–6].

Several terms have been employed to refer to this problem, such as “Internet Addiction Disorder”, “Compulsive Internet Use”, “Pathological Internet Use”, or the more common “Internet Addiction” [7–10]. While this latter term does not appear as such in any diagnostic manual (ICD-10 / DSM-IV) [11, 12], due to its relative novelty, several studies have likened the attraction of the Internet to the reinforcing properties of addictive substances [7, 13], describing symptoms or psychological profiles derived from excessive Internet use which are similar to those produced in addiction to substances (such as stress or distress due to the impossibility of connecting). In the fifth edition of the Diagnostic and Statistical Manual of Mental Disorders (DSM-V), the criteria for this condition are limited to “Internet Gaming Disorder” and do not include general use of the Internet, online gambling, or social media [14–16].

In spite of this, a recent study conducted in European adolescents found an association of this problem with other addictive behaviors, such as alcohol, tobacco, and other illegal drugs use [17]. Moreover, some authors warn of the possible association with both physical and psychological problems (lack of sleep, headaches, backaches, depression, or anxiety) [18–21].

In 1998, Kimberly Young developed the Internet Addiction Test (IAT) to assess possible dependence or addiction to the Internet [7]. This questionnaire initially consisted of 8 items based on DSM-IV diagnostic criteria for pathological gambling, but has undergone subsequent modifications in order to adapt it even further to the possible problems or behaviours related to Internet use [22, 23].

Kimberly Young’s definitive questionnaire contains 20 items and it assesses the extent to which Internet use affects daily life, social life, productivity, sleep, and the individual’s feelings. However, despite its widespread use in several languages (English, Chinese, French, Finnish, German, Italian, Greek, Arabic, and Portuguese) [23–31], some authors have questioned the quality of its psychometric properties and its capacity to distinguish between “addicts” and “non-addicts” based on the established cut-off points [32].

The IAT was developed as a unidimensional instrument to evaluate Internet Addiction but different validations have showed a factor structure with two [27, 29, 32, 33], three [24, 30, 34, 35], or six dimensions [23].

In Spain, this questionnaire has been translated by Estevez et al. [36] without having carried out a validation process with psychometric analysis. Puerta - Cortés et al. [34] evaluated its factor structure in a sample of Colombian Internet users, finding three dimensions (Consequence of Internet use, cognitive and emotional factors, and time control).

The methodological differences in all these validations (sample size, type of population sampled, the statistical method used, etc.) make it difficult to establish a comparison or know what is the best factor solution.

Moreover it should be noted that this questionnaire was developed in 1998, so some items may be outdated, given the evolution of new technologies in recent years. Authors like Pawlikowsky et al. [33], Chang & Law [24], or Lai et al. [37] established short versions of the IAT with good properties of 12 and 18 items respectively.

Therefore, the aim of this study was to validate a Spanish version of the IAT and analyse its psychometric properties using factor analysis techniques. The specific goals were: 1) to adapt to Spanish one version of the IAT using translation-back-translation method with a sample of college students, 2) to assess its reliability; and 3) to analyse its dimensional structure using exploratory, and confirmatory factor analysis.

## Methods

### Design and study population

Our study was performed in three different phases, in which we used different samples of college students. In the first phase, the adaptation and translation of IAT into Spanish, was carried out using one sample of 50 volunteer students from University of León in order to detect semantic problems. In the second phase, test-retest reliability was analysed using one sample of 80 student volunteers from the University of León who used the social networks Facebook and Tuenti. Eighteen participants were eventually excluded from the analysis due to lack of responses on some of the items. Finally, in the third phase, we performed a process of cross validation with data collected between October 2012 and January 2013 from 851 first year students participating in the uniHcos project (dynamic cohort of college students for the study of drug and other addictions), enrolled for the first time at the universities of Granada, Huelva, Jaén, León, Salamanca, and Vigo [38]. A split half – sample approach was used to evaluate structural factor test [39]. One random half was used as “development” sample (424 participants with 8 % of Problematic Internet use, 33 % males with 21 ± 6 years and 67 % females with 20 ± 4 years). The other half-sample was used as a “validity” sample (427 participants with 7 % of Problematic Internet use, 28 % males with 21 ± 5 years and 72 % females with 20 ± 4 years).

### Measurements

The IAT consists of 20 items which are scored using a five-point Likert scale (see Additional file 1), and it is designed to measure the frequency with that problematic situations arise as a result of Internet use. The test identifies two main groups of users according to the score obtained: 1) Normal users or users without problems (<40 points), and 2) problematic internet users (≥40 points) [32].

Questionnaires were administered and completed online and data were collected concerning socio demographic variables and Internet use profiles (IAT questionnaire, total time spent online per week, for leisure, or work/study. The time was measured in hours a week).

### Ethical issues

Previously, each participant completed online a written informed consent accepting the conditions of the study. The SphinxOnline® platform used in this study allows keep the confidentiality of data and thus comply with the regulations of Data Protection Act 15/1999 [40], creating two independent databases codified (one with personal data and the other one with the questionnaire data), so that no researcher can know who corresponds each questionnaire.

All Ethics Committees of the collaborating universities evaluated and accepted this procedure (Ethic Committee of University of Granada, Ethic Committee of University of Huelva, Ethic Committee of University of Jaén, Ethic Committee of University of León, Ethic Committee of University of Salamanca and Ethic Committee of University of Vigo) and the participants collaborated voluntarily without any compensation for this.

### Procedure

The questionnaire was translated into Spanish and then back-translated with the help of two native English speakers. One of them translated the test from English into Spanish, and then the other one translated the Spanish version back into English (back-translation).

This new English version was independently assessed by both individuals, comparing it with the original English version of the questionnaire in order to detect errors in reading or understanding. The Spanish version was tested on the sample of students from the University of León, in order to detect possible problems. The International Test Commission Guidelines for test adaptation were followed in all process [41].

Reliability of test-retest scores was analysed, using a between-test interval of approximately a week in order to avoid response recall [42]. The structural dimension of the IAT was analysed with two different samples of college students using exploratory and confirmatory factor analysis. As an initial analysis/estimation step, the model was fit to a “development” half-sample, with multiple model specifications. Then, the most interpretable model was chosen from among the best – fitting models, and this model’s specifications were fit to the “validation” half-sample, with modification indices used in a post-estimation examination of these model specifications [39].

### Statistical analyses

In the phase of test – retest, correlation between the two tests was analysed using Pearson’s linear correlation coefficient, whilst agreement was analysed using the Bland & Altman technique [43], employing the IAT score quantitatively, and the Kappa technique [44], comparing users without problems (<40 points) and users with problems (≥40 points) according to the score obtained in the IAT. For these analyses, we used the statistical package MedCalc v.12.3.0.

To investigate the factor structure, an Exploratory Factor Analysis (EFA) was carried out in the “development” sample. The suitability of the data for factor analysis was tested using the Kaiser-Meyer-Olkin (KMO) measure of sampling adequacy and Bartlett’s test of sphericity. The relationships between the observed variables were evaluated with the matrix of polychoric correlations due to the ordinal scale of the IAT [45]. The multivariate normality was tested and violated in the data, for this reason, we decided used a robust method to estimate the factor analysis, the Weighted Least Square Mean and Variance Adjusted (WLSMV) [46]. The factors were extracted through visual examination of a scree plot in combination with the conventional cut-off of eigenvalues greater than one or Kaiser criterion. In order to distinguish underlying constructs, Geomin oblique rotation was employed to determine factor loadings. Items were assigned to the factor that produced the highest factor loading (cut off ≥ 0.300) [47]. Goodness-of-fit was tested with chi-square (a non-significant value corresponds to an acceptable fit). However, the chi-square increases with sample size and model complexity and therefore this test was complemented by other indices that depend on a conventional cut-off: the Root Mean Squared Error of Approximation (RMSEA) and the Standardised Root Mean Square Residual (SRMR). The combination of these indices is valuable because the RMSEA is sensitive to the misspecification of the factor “loadings” and the SRMR is sensitive to the misspecification of the “co-variances”. An RMSEA between 0 and 0.05 indicates a good fit while one between 0.05 and 0.08 indicates an acceptable fit. An SRMR between 0 and 0.05 indicates a good fit and one between 0.05 and 0.10 an acceptable one. Also, the Comparative Fit Index (CFI) and Tucker Lewis Index (TLI) were used. Values in these fit indices higher than 0.90 are generally interpreted as an acceptable fit [48]. The internal consistency of each factor was confirmed by calculating Standardized Cronbach’s alphas. For each items, the corrected item-total correlation and Alpha without item were calculated [49].

Confirmatory Factor Analysis (CFA) was conducted to test the fit of our factor structure in the other half-sample, using WLSMV estimator and the same indices explained before. Also, the Weighted Root Mean Square Residual (WRMR) was calculated to compare other models.

Lastly, in order to test relationships between the established dimensions as well as with risk variables such as age and time spent online, Pearson’s correlation coefficients were calculated [46].

For all this, SPSS 20, STATA 13 and MPLUS 7.3. statistical packages were used.

## Results

Following administration of the Spanish translation of the IAT to student volunteers from the University of León, some minor modifications of a semantic nature were made to the questionnaire in order to obtain the final version (see Additional file 2). The two main changes were in item 10, *“¿Con qué frecuencia enmascaras tus problemas de la vida real con pensamientos relajantes sobre Internet?”* (How often do you block out disturbing thoughts about your life with soothing thoughts of the Internet?) using the word “*relajantes*” instead of “*tranquilizantes*”, and in item 20*, “¿Con qué frecuencia te sientes deprimido, de mal humor o nervioso cuando no estás conectado y se te pasa todo en cuanto vuelves a conectarte?”* (How often do you feel depressed, moody, or nervous when you are off-line, which goes away once you are back on-line?), using the term “*de mal humor*” instead of the initial word “*temperamental*”.

In the reliability test-retest scores analysis, the retest was conducted after an average of 8.3 ± 3.1 days (range: 5–17 days; Median = 7.0 days). The total mean score obtained in the first test was 17.8 ± 11.5 points (range = 0–52, median = 15.5), and in the second test the mean score was 14.2 ± 11.1 points (range = 0–48, median = 12.0).

**Fig. 1:**
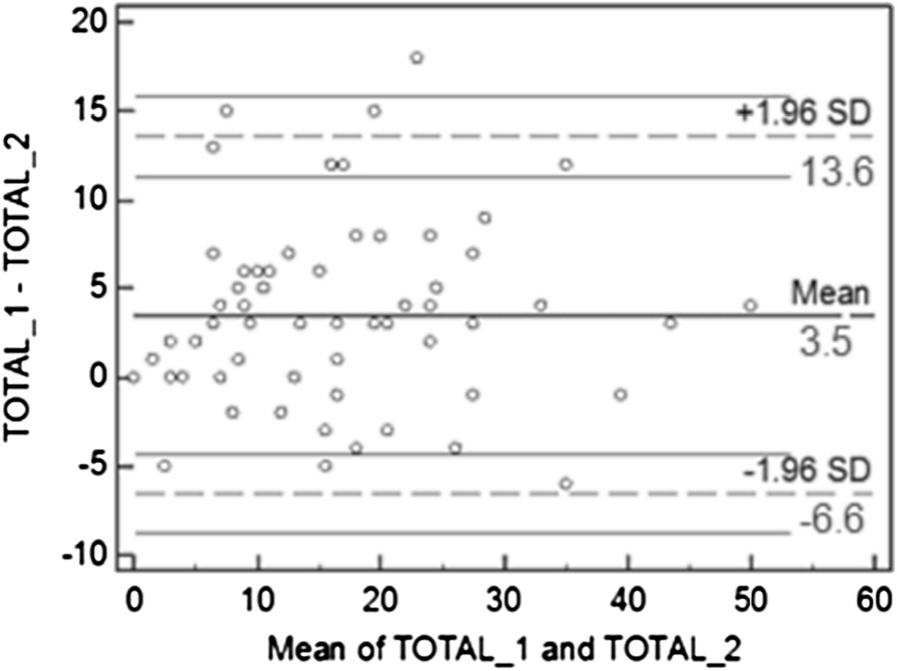
Data analysis using the Bland-Altman technique. The terms used refer to the following: Total 1 = Total score obtained in the first test; Total 2 = Score obtained in the second test; Mean = Average, SD = Standard Deviation

The correlation obtained between the total scores of both tests was good (*r* = 0.899), as was agreement according to the Bland-Altman technique (Fig. 1), with a mean difference of 3.5 and a 95 % CI between −6.6 and 13.6 when a quantitative analysis was conducted of the IAT score in the two tests. A good agreement was also obtained for the detection of users with problems (0.650) according to the Kappa technique.

In the preliminary analysis of “development” half-sample of 424 college students, the Kaiser-Meyer-Olkin index was 0.924, and the Bartlett test of sphericity was significant (*χ*2 = 3440.21; df = 190; *p* < 0.001), showing the adequacy of the data for use in the EFA.

One first analysis showed that three factors could be extracted. However, in the third factor only there was one variable with load higher than 0.300 and the Item_7 did not load in any factor. After that, we decided remove this last variable and repeat the EFA. Then, two factors were extracted (correlation 0.654; *p* < 0.001) that explained 55 % of the total variance (Table 1), yielding a Cronbach’s alpha for the questionnaire as a whole of 0.91 and showing acceptable fit indices: RMSEA = 0.063 (90 % IC 0.055 – 0.071); CFI = 0.921; TLI = 0.957; SMRM = 0.049.

**Table 1:** Items corresponding to each factor after the exploratory analysis

Item	Mean	SD	Skewness	Kurtosis	Corrected item-total correlation	Alpha without item	Factor loading	
							Factor 1^a^	Factor 2^b^
20	0.29	0.724	3.161	14.524	0.608	0.897	**0.863**	−0.008
10	0.56	1.066	2.332	8.511	0.601	0.896	**0.774**	0.035
13	0.57	0.912	1.942	7.245	0.598	0.896	**0.762**	−0.007
11	0.69	0.967	1.682	6.212	0.627	0.896	**0.734**	0.071
15	0.50	0.845	1.892	6.487	0.664	0.895	**0.723**	0.132
12	0.80	1.126	1.555	5.063	0.531	0.898	**0.688**	−0.001
19	0.31	0.717	2.774	11.363	0.396	0.901	**0.685**	−0.066
3	0.21	0.667	4.275	24.174	0.310	0.902	**0.676**	−0.127
9	0.63	1.021	1.963	6.953	0.562	0.897	**0.541**	0.213
14	1.12	1.290	1.161	3.750	0.616	0.895	**0.423**	0.346
4	1.00	1.112	1.183	4.279	0.340	0.903	**0.347**	0.093
8	0.86	1.142	1.395	4.375	0.611	0.895	−0.021	**0.844**
6	0.99	1.137	1.153	3.899	0.660	0.894	0.003	**0.841**
17	0.96	1.216	1.203	3.677	0.597	0.896	0.124	**0.689**
1	2.34	1.412	0.172	2.120	0.474	0.900	−0.035	**0.629**
2	1.43	1.242	0.780	3.102	0.614	0.895	0.139	**0.622**
16	1.57	1.351	0.547	2.460	0.617	0.895	0.261	**0.533**
18	0.45	0.903	2.732	11.451	0.636	0.896	0.404	**0.469**
5	0.92	1.108	1.203	3.928	0.559	0.897	0.331	**0.379**
Standarized alpha							0.86	0.86
Eigenvalue							9.25	1.57
Total explained variance							46.96 %	7.86 %

The higher of the two-factor loadings are printed in bold. All of these were significant at 5 % level

^a^Factor 1 = emotional investment

^b^Factor 2 = time management and performance

Factor 1 (called “Emotional investment”) explained 47 % of the total variance and consisted of eleven items related to the feelings that an individual experiences when unable to connect to the Internet, or the attitude an individual adopts when asked what he or she does while online. Factor 2 (called “Time Management and Performance”) explained 8 % of the total variance and consisted of 8 items related to possible impairment of academic or professional performance and excessive time spent online.

**Fig. 2:**
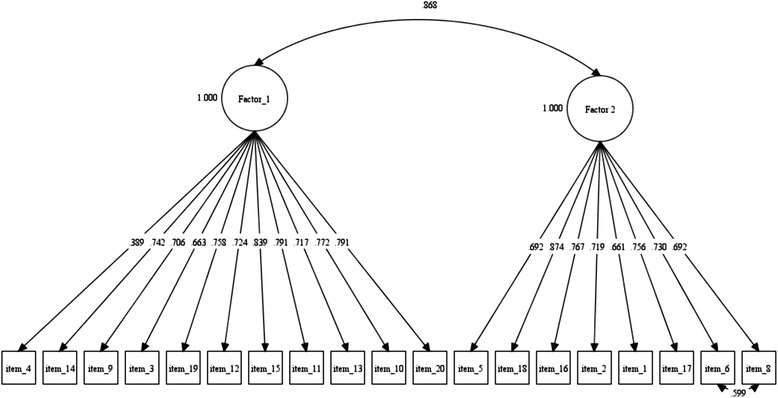
Confirmatory Factor Analysis of IAT. Items 6 and 8 were covariate due to their semantic similarity. Factor_1 = Emotional Investment; Factor_2 = Time Management and Performance. All standardized estimations were significant at level 5 %

After that, four models were tested using CFA in the “validation” half-sample of 427 college students (Table 2). Model 1 with one dimension of 20 original items of the IAT, the model 2 as one dimensional but with 19 items (without item 7), the model 3 with the two dimensions detected in the EFA, and the model 4 incorporating a small modification, co-varying items 6 and 8, given their possible semantic similarity and the recommendations of modification indices, obtaining a better fit of the model (Fig. 2).

**Table 2:** Comparison of goodness of fit indices obtained from the confirmatory factor analysis

	Items	Factors	*χ*2	df	*χ* ^2^/df	RMSEA (90 % CI)	CFI	TLI	WRMR
Model 1	20	1	794.128^**^	170	4.7	0.093 (0.086 - 0.099)	0.914	0.904	1.451
Model 2	19	1	775.386^**^	152	5.1	0.098 (0.091 - 0.105)	0.912	0.901	1.482
Model 3	19	2	594.173^**^	151	3.9	0.083 (0.076 - 0.090)	0.938	0.929	1.272
Model 4	19	2	451.741^**^	150	3.0	0.069 (0.061 - 0.076)	0.958	0.952	1.081

^**^ *p* < 0.001

In relation to the other variables (Table 3), both factors correlated negatively with age and positively with hours spent online per week in total and for leisure purposes. No statistically significant differences were found between any of the dimensions and time spent online for academic or professional reasons.

**Table 3:** Correlation between IAT factors with other variables

	Factor 1	Factor 2
	Emotional investment	Time management and performance
Age^a^	−0.121^*^	−0.170^*^
Total Time^b^	0.322^**^	0.310^**^
Leisure Time^b^	0.346^**^	0.334^**^
Work/Study Time^b^	0.010	−0.002

^a^Age in years

^b^Hours per week

^*^ *p* < 0.05

^**^ *p* < 0.001

## Discussion

The Spanish version of the IAT reported here has shown good evidence of reliability and acceptable psychometric properties for use in the analysis of problems related to Internet use and abuse, demonstrating a positive correlation with time spent online, especially for leisure purposes.

A wide range of diagnostic tools have been used in previous studies examining pathological Internet use [7, 8, 50–55], the majority of which are closely related to the criteria established in the DSM – IV [12] and the ICD-10 [11] for pathological gambling or substance addiction. However, most of the questionnaires used to detect potentially problematic or addictive behaviours related to Internet use have been based on national surveys or ad hoc questionnaires that have not been previously validated [56].

According to Chang, 2008 [24], regardless of the type of instrument used, when the dimensionality of problematic Internet use is analysed, it is possible to find common aspects that can be grouped into more or fewer dimensions in the factorial solutions, as: 1) Compulsive Internet use and excessive time spent online, 2) withdrawal symptoms when being restricted from Internet use, 3) Using online social interaction to replace Real-life interpersonal activities, and 4) Negative consequences related to Internet use (as social, academic or work problems).

Of all the questionnaires found in the current scientific literature to evaluate this problematic, the most widely used is the one created by Kimberly Young [7], whose psychometric properties have shown the existence of both unidimensional and multidimensional solutions [22–32, 37].

For this reason, we decided to adapt this questionnaire in Spanish and evaluate its psychometric properties following a strict methodology according to the latest recommendations [45–47].

The Spanish version of Puerta-Cortés et al. [34] showed similarities in the context of the questions with our results, but the translation of sentences is somewhat different, given the cultural differences between Spanish and Colombian population.

As part of our validation process, reliability test-retest scores was assessed using a mean between-test interval of 8.3 ± 3.1 days, within the range recommended in the literature [44], obtaining a level of reliability (*r* = 0.899) very similar to that reported in the initial validation of the IAT (*r* = 0.85) [32]. Our results also showed good agreement using both the Kappa method (0.650) and Bland-Altman technique (mean difference 3.5), aspects that cannot be compared for lack of these parameters in other validations.

The estimation of internal consistency was analysed using Cronbach’s alpha, obtaining a value (0.91) which was similar to that found in other published validations, in which the value has ranged from 0.89 to 0.93 [25–27].

Moreover, our analysis of the questionnaire’s dimensions revealed the existence of two principal factors (“Emotional Investment” and “Time management and Performance”) which explained 55 % of the variance, consistent with other published psychometric analyses, independently of the number of factors reported that varied between one and six depending on the characteristics of the samples used for the analyses [23–32].

Chinese version conducted in 2008 by Chang and Law [24], reported the existence of three factors explaining 57 % of the variance. The first, which they called “Withdrawal and Social Problems”, was related primarily to alterations in behaviour when the subject was not online and to thoughts of what he or she will do the next time they connect to the Internet, corresponding to what we have called “Emotional Investment”. The second dimension identified by these authors coincides with our second factor, “Performance and Time Management”, which encompasses issues related to time spent online and how a loss of control in this aspect can impair academic and professional performance. Lastly, the third component described in the Chinese study was called “Reality Substitution” and consisted of items related to the social relationships established through the Internet and a preference for these as opposed to relationships in real life, in turn related to our first factor [24].

On the other hand, an analysis of the IAT’s factor structure conducted by Widyanto and McMurran [23] indicated the existence of six factors (salience or preoccupation about what will be done during the next session online, excessive use, neglecting work, anticipation, lack of control, and neglecting social life) which explained a total of 68 % of the variance. However, these same authors conducted a later psychometric study [35], in which they found that these six components could be grouped into three main dimensions that explained 56 % of the variance. In this later study, they established the following dimensions: “Psychological emotional conflict”, “mood modification” (consistent with our first factor), and “time management” (similar to our second factor).

Another important validation study was that conducted of the French version by Khazaal et al. [25], who performed a factor analysis comparing the six-factor model [23] described in the previous paragraph with a single-factor model. This latter solution explained 45 % of the variance and resulted in better psychometric properties compared to the six-factor model.

Lastly, one of the most recent psychometric analysis, conducted in Germany [27], showed the existence of two factors (preoccupation and loss of control), explaining between 63 and 73 % of the variance depending on whether the questionnaire was administered in paper or online format.

Among all the factors found in different studies, time spent online is highlighted as a benchmark, due to it is an indicator of problems related to Internet use. However, it is not a single criterion, reason why in different psychometric analyses other aspects as lack of self-control, negative consequences in daily life related to internet use or reality substitute among others, have been referred to as related to it [24].

Furthermore, our adaptation of the IAT to Spanish showed that some item of the questionnaire might be outdated. This problem was found by other authors, who have validated short versions of the IAT with 12 and 18 items [29, 33]. Costello & Osborne [47] suggest that problematic items (ones that are low-loading, crossloading or Freestanding) must be removed of the factor analysis. In the first EFA conducted in this paper, the Item_7 did not have enough loading on any factor (factor loadings lower than 0.300), reason why was eliminated. We removed this item considering also a theoretical explanation based in other validations [24, 37], due to the IAT questionnaire was development in 1998 [7], when the email was checked by computer, but today, we can see the email in our smartphones many times a day and this behaviour can be considered normal. For this, we believe that this item was not relevant to evaluate the Problematic Internet Use and it did not compromise the validity of the questionnaire.

In addition, the covariation between item_6 and item_8 increased the fit model in the confirmatory factor analysis. This covariation was done according to the modification fit indices values, to other previous validations of the IAT and to the content similarity semantic of the items [31, 37, 46].

Although pathological use or addiction to the Internet is not recognised as such in any diagnostic manual [11, 12], some authors have underscored excessive time spent online and age as risk variables [24, 35]. For this reason, we analysed the possible relationship with these variables, obtaining results similar to those reported by Widyanto et al. for the English version of the IAT [23], indicating a negative relationship with age and a positive one with time spent online, especially for leisure and entertainment purposes.

With respect to the main limitations of this study, we emphasize that the sample used is voluntary and nonrandomized, as well as being college population, which hinders the generalizability of the results. Moreover, the study presents selection and information biases due to the online methodology used in the development of questionnaires. Finally, methodological differences with other published studies published (type of population, statistic method used, sample size, etc.) make difficult to conduct a thorough data comparison.

On the other hand, our main strength is the strict methodological analysis used. We have evaluated the association between variables using polychoric correlations (given the ordinal nature of the IAT scale) instead of Pearson correlations [45]. Moreover, as the sample size was large enough we used a split-half sample approach, using a subsample to EFA and the other one to CFA [39]. And finally, given the violation of normality in our data, we used robust statistical methods to the analyses [46].

For all these reasons, we believe that the psychometric properties of our short version of IAT presents acceptable characteristics to use it as a diagnostic or screening tool of Internet related problems in daily life, considering as future lines of research the influence of gender in these problems or the evaluation of psychological diseases associated, as well as the analyse in general population to improve the external validity.

## Conclusions

The two main dimensions were founded in our validation of the IAT: “emotional investment” and “Time Management and Performance” which correlated negatively with age and positively with hours spent online per week. This Spanish short version of the IAT showed good properties to evaluate problematic Internet use in college students.

## Additional files

Additional file 1:
**English version of IAT.** (DOC 39 kb) Additional file 2:
**Spanish version of IAT.** (PDF 7 kb)

## Abbreviations

CFA: Confirmatory Factor Analysis
CFI: Comparative Fit Index
DSM-V: Diagnostic and Statistical Manual of Mental Disorders 5^th^ edition
EFA: Exploratory Factor Analysis
IAT: Internet Addiction Test
KMO: Kaiser-Meyer-Olkin
RMSEA: Root Mean Squared Error of Approximation
SRMR: Standardised Root Mean Square Residual
TLI: Tucker Lewis Index
WLSMV: Weighted Least Square Mean and Variance Adjusted
