# Supplementary material for: Validation and psychometric analysis of the Internet Addiction Test in Spanish among college students
Source: BMC Public Health. 2015 Sep 24;15:953. doi: 10.1186/s12889-015-2281-5 (PMC4581075; doi:10.1186/s12889-015-2281-5)
Supplement: Additional file 1: — English version of IAT. (DOC 39 kb) [file 12889_2015_2281_MOESM1_ESM.doc]

**Additional file 1**. English version of IAT.

|  | Item | Question |  |
| --- | --- | --- | --- |
|  | 1 | How often do you find that you stay on-line longer than you intended? |  |
|  | 2 | How often do you neglect household chores to spend more time on-line? |  |
|  | 3 | How often do you prefer the excitement of the Internet to intimacy with your partner? |  |
|  | 4 | How often do you form new relationships with fellow on-line users? |  |
|  | 5 | How often do others in your life complain to you about the amount of time you spend on-line? |  |
|  | 6 | How often do your grades or school work suffer because of the amount of time you spend on-line? |  |
|  | 7 | How often do you check your e-mail before something else that you need to do? |  |
|  | 8 | How often does your job performance or productivity suffer because of the Internet? |  |
|  | 9 | How often do you become defensive or secretive when anyone asks you what you do on-line? |  |
|  | 10 | How often do you block out disturbing thoughts about your life with soothing thoughts of the Internet? |  |
|  | 11 | How often do you find yourself anticipating when you will go on-line again? |  |
|  | 12 | How often do you fear that life without the Internet would be boring, empty, and joyless? |  |
|  | 13 | How often do you snap, yell, or act annoyed if someone bothers you while you are on-line? |  |
|  | 14 | How often do you lose sleep due to late-night log-ins? |  |
|  | 15 | How often do you feel preoccupied with the Internet when off-line, or fantasize about being on-line? |  |
|  | 16 | How often do you find yourself saying "just a few more minutes" when on-line? |  |
|  | 17 | How often do you try to cut down the amount of time you spend on-line and fail? |  |
|  | 18 | How often do you try to hide how long you've been on-line? |  |
|  | 19 | How often do you choose to spend more time on-line over going out with others? |  |
|  | 20 | How often do you feel depressed, moody, or nervous when you are off-line, which goes away once you are back on-line? |  |
| Scale: 0 = Not Applicable; 1 = Rarely; 2 = Occasionally; 3 = Frequently; 4 = Often; 5 = Always. | | | |
